# Supplementary material for: Regulating electron configuration of single Cu sites via unsaturated N,O-coordination for selective oxidation of benzene
Source: Nat Commun. 2022 Nov 16;13:6996. doi: 10.1038/s41467-022-34852-y (PMC9668809; doi:10.1038/s41467-022-34852-y)
Supplement: Supplementary file 1 — Supplementary Information [file 41467_2022_34852_MOESM1_ESM.pdf]

# Supplementary Information

# Regulating electron configuration of single Cu sites via unsaturated N,O-coordination for selective oxidation of benzene

Ting Zhang,[1] Zhe Sun,[1] Shiyan Li,[2] Baojun Wang,[3] Yuefeng Liu,[2,*] Riguang Zhang,[3,*] and Zhongkui Zhao[1,*]

[1]State Key Laboratory of Fine Chemicals, Department of Catalysis Chemistry and Engineering, School of Chemical Engineering, Dalian University of Technology, Dalian 116024, P. R. China

[2]Dalian National Laboratory for Clean Energy (DNL), Dalian Institute of Chemical Physics, Chinese Academy of Science, Dalian 116023, P. R. China

[3]State Key Laboratory of Clean and Efficient Coal Utilization, Taiyuan University of Technology, Taiyuan 030024, P. R. China; College of Chemical Engineering and Technology, Taiyuan University of Technology, Taiyuan 030024, P. R. China

**Corresponding Author:** *Zhongkui Zhao, E-mail: zkzhao@dlut.edu.cn. *Yuefeng Liu, E-mail: yuefeng.liu@dicp.ac.cn.*Riguang Zhang, Email: zhangriguang@tyut.edu.cn

**Supplementary Methods.**

**Chemicals.** Melamine, copper nitrate hydrate ($Cu(NO_3)_2 \cdot 3H_2O$), ferric nitrate ($Fe(NO_3)_3 \cdot 9H_2O$), cyanuric acid, benzene, acetonitrile ($CH_3CN$), *n*-tetradecane and dichloromethane ($CH_2Cl_2$) were purchased from Tianjin Fuyu Fine Chemical Co., Ltd. Dimethyl sulfoxide (DMSO), Copper(II) acetate monohydrate ($Cu(OAc)_2 \cdot H_2O$) and $H_2O_2$ (30%) were obtained from Damao Chemical Reagent Factory. Polyvinylpyrrolidone (PVP-K30) and potassium thiocyanate (KSCN) were purchased from Sigma. Sodium borohydride ($NaBH_4$) and sodium hydroxide (NaOH) were purchased from Tianjin Guangfu Technology Development Co., Ltd. Deionized water was home-made. All of the chemicals were analytical grade and used directly without further purification.

**Characterization.** FTIR spectra were collected in the wavenumber range of 4000-400 $cm^{-1}$ on EQUINOX-55 Fourier Transform Infrared Spectrometer (BRUKER). SEM images were recorded by a FEI QUANTA 450 scanning electron microscopy. The TEM and HAADF-STEM images were collected on a JEOL-2100F FETEM with 200kV of electron

acceleration energy. XRD patterns were recorded with an X-ray diffractometer equipment of Rigaku Corporation SmartLab 9 using Cu *K*α radiation. XPS spectra were recorded on an Al *K*α radiated Thermo VG ESCALAB250 instrument. The binding energy (BE) was calibrated by C 1*s* peak at 284.6 eV as the internal standard, and the deconvolution of spectra were carried out using the XPS PEAK 41 program with Gaussian function after subtracted by a Shirley background. Cu content was determined by an Optima 7300 DV inductively coupled plasma atomic emission spectrometer (ICP-AES), the solid sample was disolved in concentrated $H_2SO_4$ and $HNO_3$ for ICP-AES test. Nitrogen adsorption-desorption isotherms were recorded by 3H-2000PSI system of Beishide apparatus at 77 K. A degas process was performed at 140 $^oC$ for 6 h before the test. Brunauaer-Emmeetr-Teller (BET) model was applied for the analysis of the porosity with the pore volume measured at $P/P_0$ = 0.99 point. The mesopore size distribution was calculated by BJH method from adsorption branch. XAFS spectra were corrected at the XAS station (BL14W1) of the Shanghai Synchrotron Radiation Facility (SSRF). The electron storage ring was operated at 3.5 GeV. Si(311) double-crystal was used as the monochromator, and the data was collected using solid-state detector under ambient conditions. The beam size was limited by the horizontal and vertical slits with the area of 1×4 $mm^2$ during XAS measurements. The X-ray absorption of Cu foil at Cu *K*-edge of was measured for energy calibration.The obtained XAFS data was processed in Athena (version 0.9.26) for background, pre-edge line and post-edge line calibrations. Then Fourier transformed fitting was carried out in Artemis (version 0.9.26). The $k^3$ weighting, k-range of 3-12 $Å^{-1}$ and *R* range of 1-3 Å were used for the fitting of Cu foil. k-range of 3-10 $Å^{-1}$ and *R* range of 1-3 Å were used for the fitting of Cu sample. The four parameters, coordination number, bond length, Debye-Waller factor and $E_0$ shift ($C.N.$, $R$, $\sigma^2$, $\Delta E_0$) were fitted without anyone was fixed, constrained, or correlated. For Wavelet Transform analysis, the χ(k) exported from Athena was imported into the Hama Fortran code. The parameters were listed as follow: R range, 1 - 4 Å, k range, 0 - ~12 $Å^{-1}$ for standards and 0 - 10 $Å^{-1}$ for sample; k weight, 2; and Morlet function with $\kappa$=10, $\sigma$=1 was used as the mother wavelet to provide the overall distribution.

**Comparison of $H_2O_2$ activation.** The test was performed at 60 $^oC$, and the system includes 10 mg of catalyst, 2 mL of $CH_3CN$ as solvent, 2 mL of benzene and 0.25 mL of $H_2O_2$ (the mole ratio of benzene/$H_2O_2$ is 10:1). The test was carried out for 0.25 h, 0.5 h, 1.0 h, and 1.5 h, respectively. The solid was removed by centrifugation, and the remained $H_2O_2$ is determined by iodometry. The turnover number (*TON*) of consumed $H_2O_2$ was calculated as (mole of consumed $H_2O_2$)/(mole of active Cu).

**Kinetic study.** The kinetic study was carried out in a temperature range of 50-80 $^oC$ within kinetic controlled region. Typically, the reaction system includes 10 mg of catalyst and 2 mL of $CH_3CN$ as solvent, 0.10 mL of substrate and 1.0 mL of $H_2O_2$ (the mole ratio of $H_2O_2$/benzene is 8:1). The reaction rate (*r*) was obtained by dividing the mole of conversed substrate by the raction time and mole of actvie Cu atoms. To simplify calculations, the reaction order is assumed to be 1. By plotting ln$k$ as a function of $1/T$, the Arrhenius plot was achieved. With the slope, the apparent activation barrier ($E_a$) was achieved.

**Supplementary Note 5. Comparison of phenol oxidation.** Typically, 20 mg of catalyst and 4 mL of $CH_3CN$ as solvent were used, then 0.2 mL of phenol with 0.5 mL $H_2O_2$ was added. After reacted at 60 $^oC$ for 1 h, the phenol conversion was determined by gas chromatograph.

**Supplementary Note 6. Recyclability of Cu-$N_1O_2$ SA/CN for benzene selective oxidation.** To test the stability of single-atom Cu-$N_1O_2$ SA/CN catalyst for benzene selective oxidation to phenol, the recycling experiment was performed. The reaction conditions are the same as above. After one cycle of reaction, solid catalyst was recovered by centrifugation, washed with ethanol and dried. The recovered catalyst was used for the next cycle.

**Supplementary Note 7. Calculation methods.** Theoretical calculations have been implemented by $Dmol^3$ code in Materials Studio 8.0 package[1,2]. To describe the exchange-correlation functional, the generalized gradient approximation (GGA-PBE) is employed[3-5]. The effective core potential (ECP) is used for Cu atoms; for other non-metal atoms, all-electron basis set is used[6]. The Brillouin zone *k*-point of 3×3×1 was used. The valence electron function is expanded using the double-numeric polarized basis set (DNP)[7,8]. For all calculations, the maximum force, maximum distance, and energy convergence are set to be $4\times10^{-3}$ Ha/Å, $5\times10^{-3}$ Å and $2\times10^{-5}$ Ha, respectively. All configurations were fully optimized in the calculations, 0.005 Hartree was used for the smearing value. The long-range dispersive force was evaluated using DFT-D approach proposed by Grimme[9]. Aiming at searching for transition states, the complete linear synchronous transit/quadratic synchronous transit (LST/QST) approach was used[10,11]. The rationality of transition states was verified by the calculations of TS Confirmation and Vibrational Analysis.

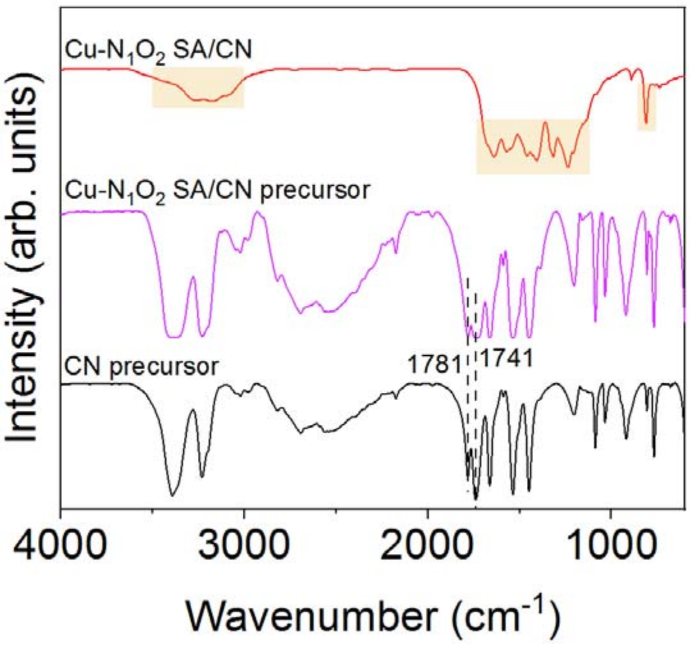


**Supplementary Fig. 1** The FTIR spectra of CN precursor, Cu-$N_1O_2$ SA/CN precursor and Cu-$N_1O_2$ SA/CN catalyst.

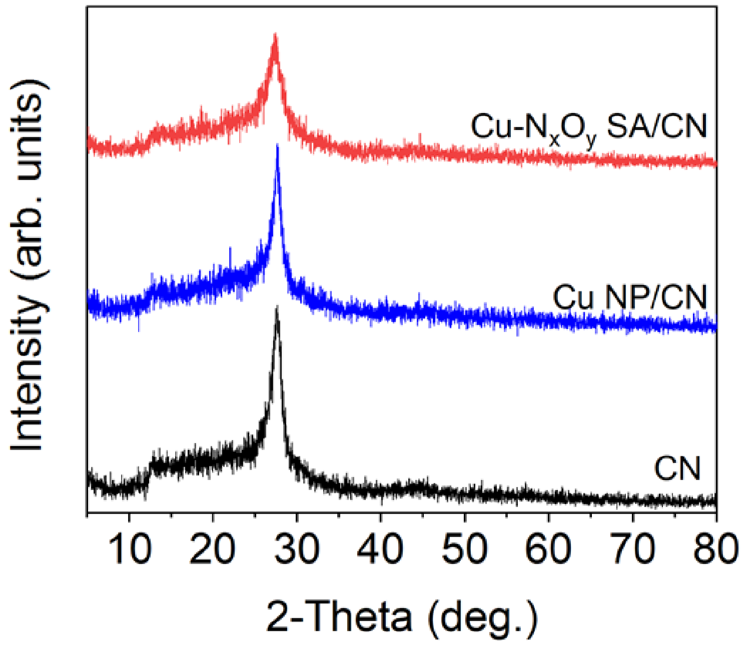


**Supplementary Fig. 2** The XRD patterns of as-prepared samples.

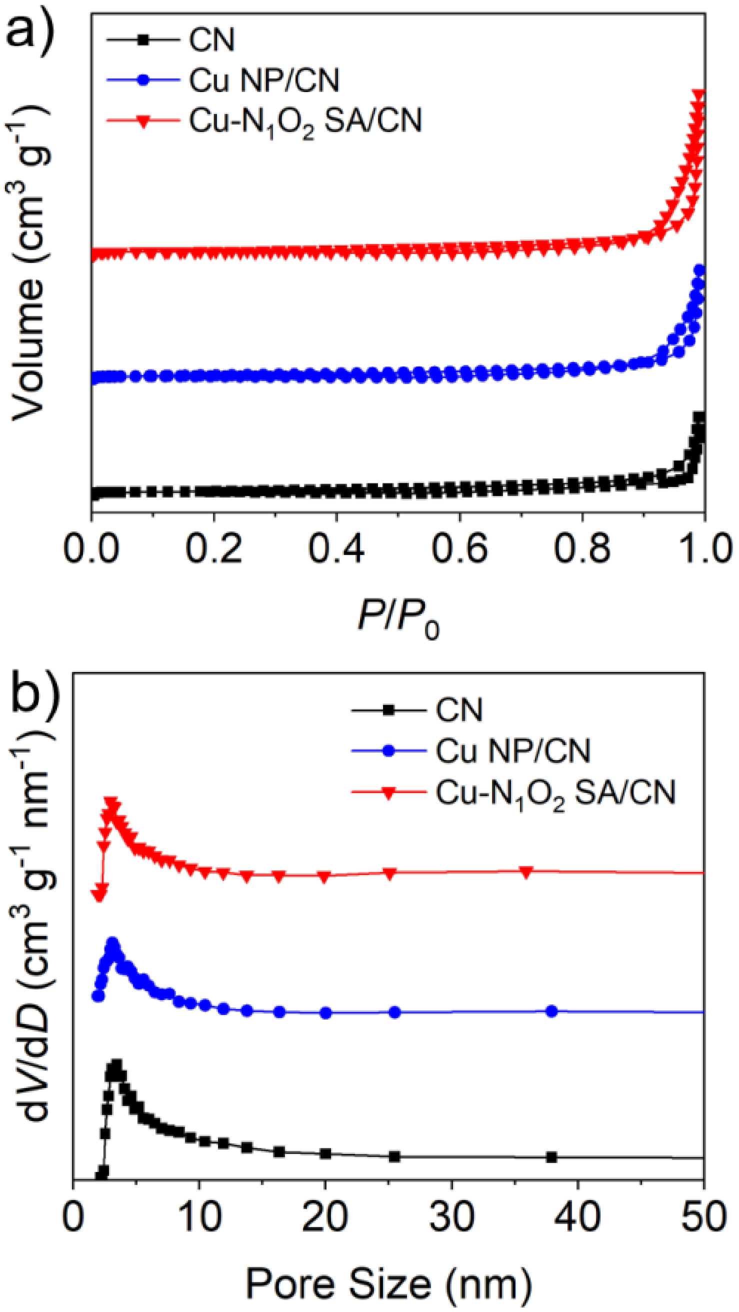


**Supplementary Fig. 3** a) $N_2$-physisorption isothermal curves and b) corresponding pore size distribution curves of as-prepared samples.

**Supplementary Table 1.** Textural properties of as-prepared samples.

| Entry | Sample | $S_{BET}$ ($m^2$ $g^{-1}$) | $V_{BJH}$ ($cm^3$ $g^{-1}$) | Pore size (nm) |
|---|---|---|---|---|
| 1 | CN | 44.6 | 0.35 | 3.1 |
| 2 | Cu NP/CN | 42.3 | 0.39 | 3.1 |
| 3 | Cu-$N_1O_2$ SA/CN | 41.1 | 0.72 | 3.0 |

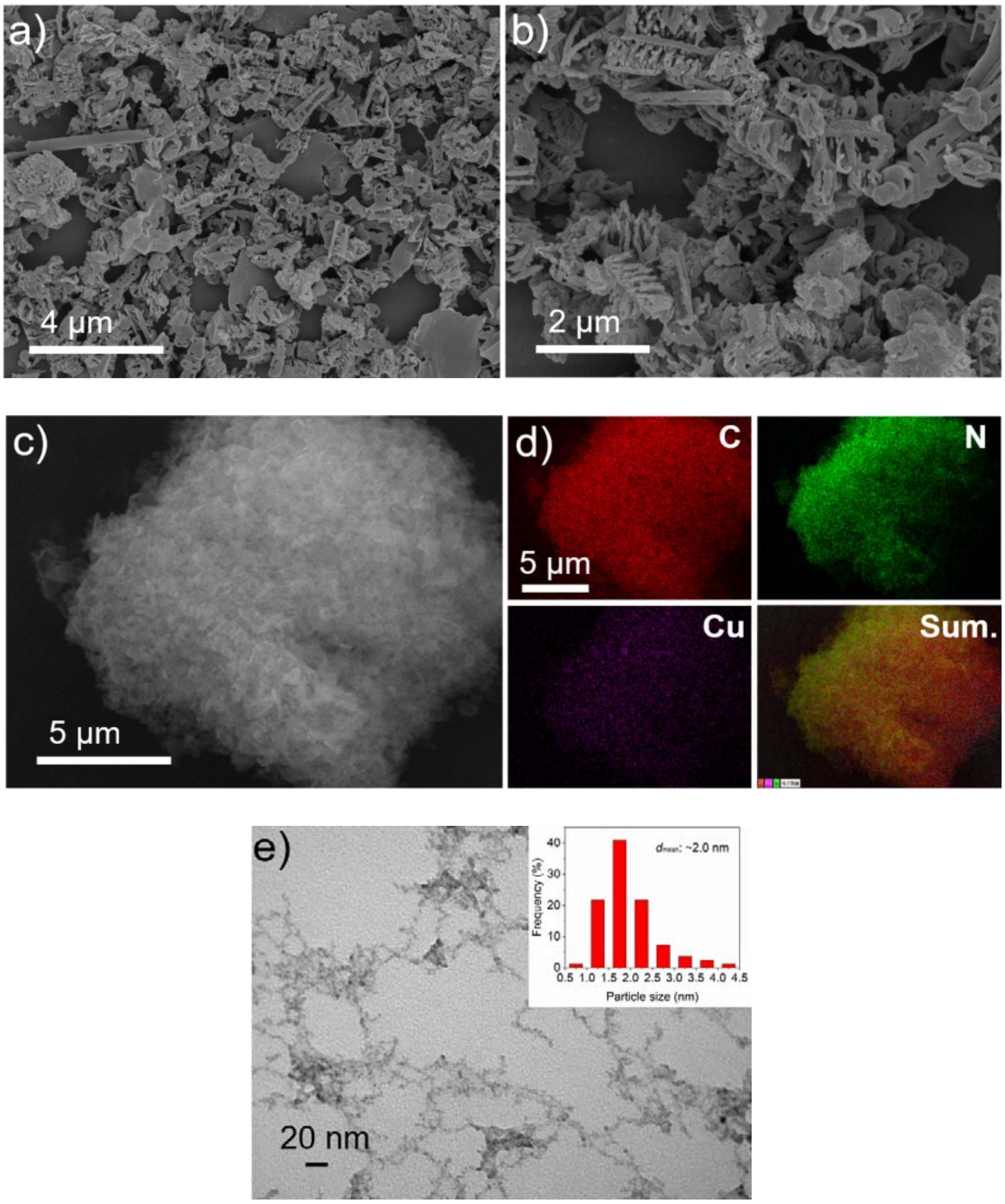


**Supplementary Fig. 4** a) SEM image of CN support, b) SEM image, c) TEM image, d) the local EDX elemental mappings of Cu NP/CN catalyst, e) The TEM image and particle distribution of Cu nanoparticles.

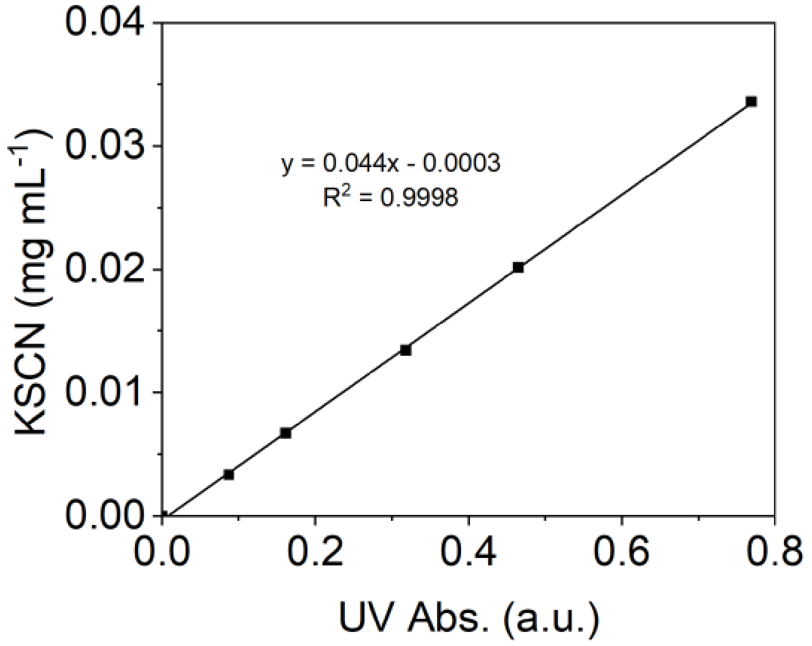


**Supplementary Fig. 5** The standard curve for KSCN titration.

**Supplementary Table 2.** Data of KSCN titration for determine the dispersity of Cu on single-atom catalysts

| Entry | sample | Mass (mg) | Cu loading (wt%)[a] | Abs. (a.u.) | Consumed KSCN (mg) | $D_{Cu}$ (%)[b] |
|---|---|---|---|---|---|---|
| 1 | $Cu-N_3$ SA/CN | 51.0 | 0.85 | 0.696 | 0.33 | 49 |
| 2 | $Cu-N_2$ SA/CN | 51.4 | 0.20 | 0.738 | 0.14 | 92 |
| 3 | $Cu-N_1O_2$ SA/CN | 51.4 | 0.16 | 0.748 | 0.10 | 79 |
| 4 | CN | 51.8 | 0 | 0.768 | 0.01 | - |

[a] Determined by ICP-AES. [b] Cu dispersity: (Mole of consumed KSCN) / (Mole of Cu)

KSCN titration was carried out as follow: 1) 0.1681 g of KSCN was dissolved in 500 mL deionized water, the obtained aqueous solution was denoted as A. 25 g of $Fe(NO_3)_3 \cdot 9H_2O$ with 12.5 mL concentrated $HNO_3$ was dissolved in 250 mL deionized water, the solution was denoted as B. 2) 5 mL of B was mixed with 0, 0.5, 1.0, 2.0, 3.0 and 5.0 mL of A, respectively, and the each mixture as diluted to 100 mL in volumetric bottle (100 mL). Then the absorbancy of the six samples were determined on an ultraviolet and visible spectrophotometer. According to the absorbancy and KSCN concentration, we can get a standard curve (Figure S5). 3) 5 mL of A was diluted to 100 mL. 50 mg of single-atom sample was added in to the aqueous solution and stirred for 12 h at room temperature. Then the solid was removed by filtration. 5 mL of B was added into the filtrate, and the absorbancy was determined. According to the remained and initial amount of KSCN, basing on the hypothesis of one Cu site absorb one $SCN^{-1}$, the amount of active Cu can be inferred (Table S2)[12,13]. Moreover, the results indicate minimal effect of CN carrier during the test.

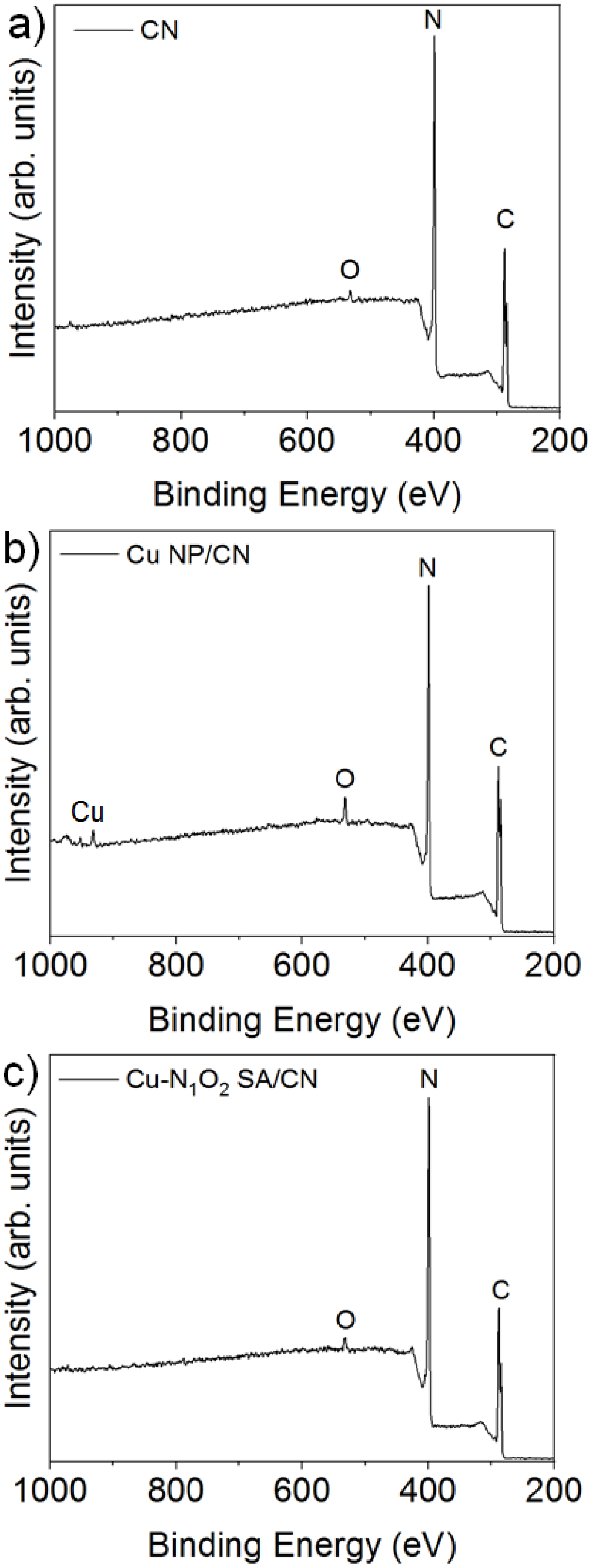


**Supplementary Fig. 6** Survey XPS spectra of a) CN, b) Cu NP/CN and c) Cu-$N_1O_2$ SA/CN.

**Supplementary Table 3.** Surface component of as-prepared samples.

| Entry | Sample | C (atomic%) | N (atomic%) | O (atomic%) | C/N |
|---|---|---|---|---|---|
| 1 | CN | 50.49 | 48.13 | 0.03 | 1.05 |
| 2 | Cu NP/CN | 51.79 | 43.81 | 3.70 | 1.11 |
| 3 | Cu-$N_1O_2$ SA/CN | 49.14 | 49.07 | 1.79 | 1.00 |

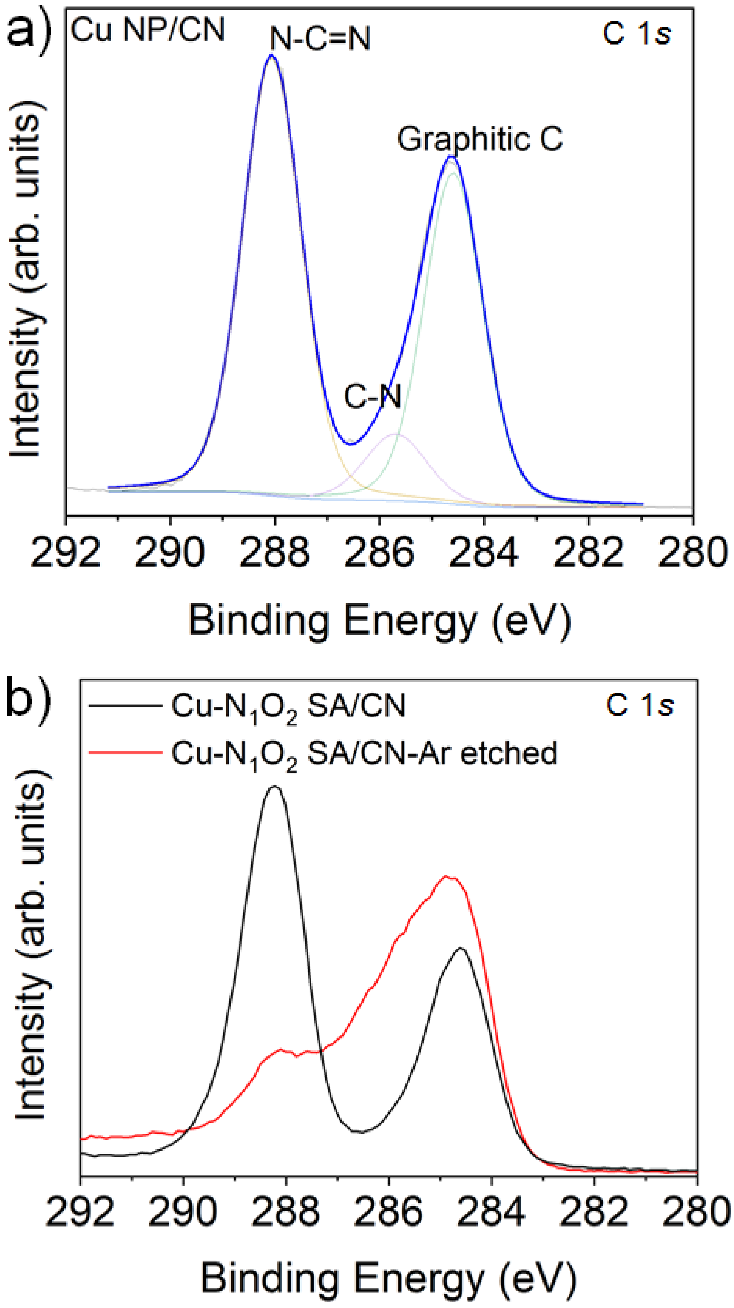


**Supplementary Fig. 7** The C 1s XPS spectra of a) Cu NP/CN and b) Cu-$N_1O_2$ SA/CN and after Ar etching for 60 s.

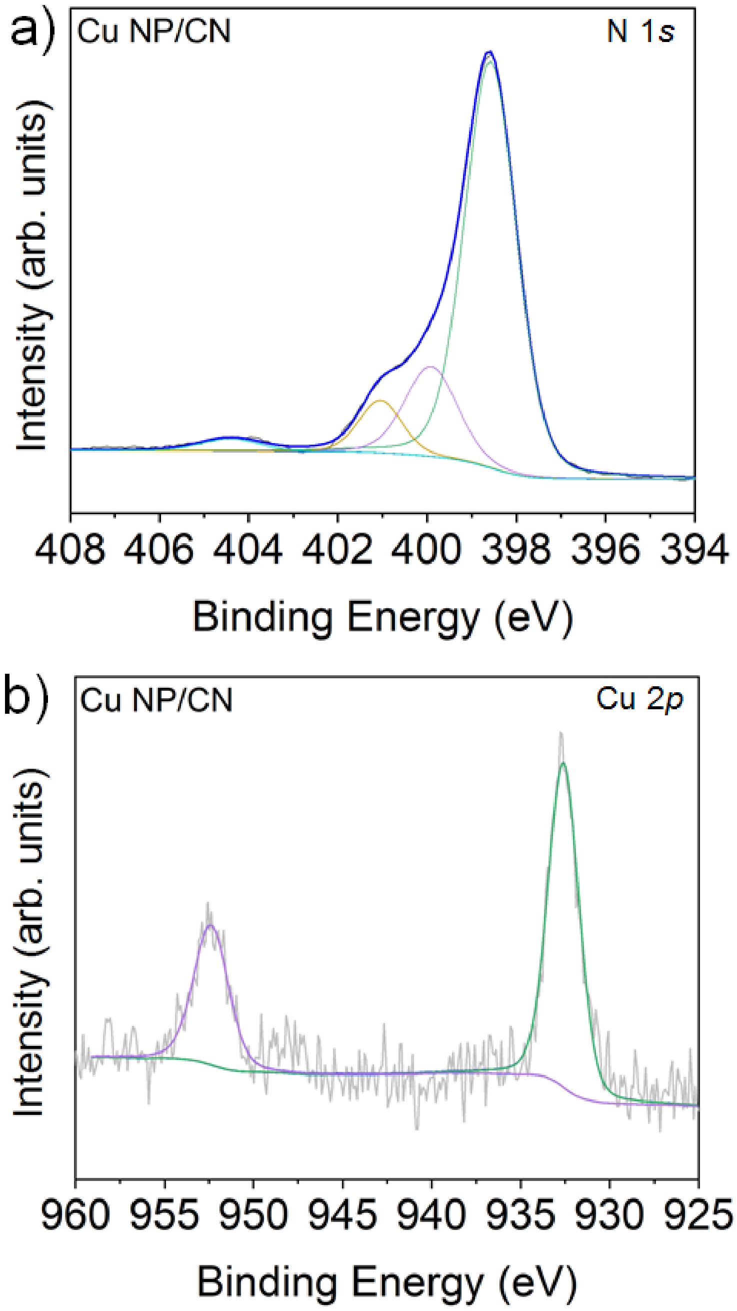


**Supplementary Fig. 8** The a) N 1s and b) Cu 2p XPS spectra of Cu NP/CN.

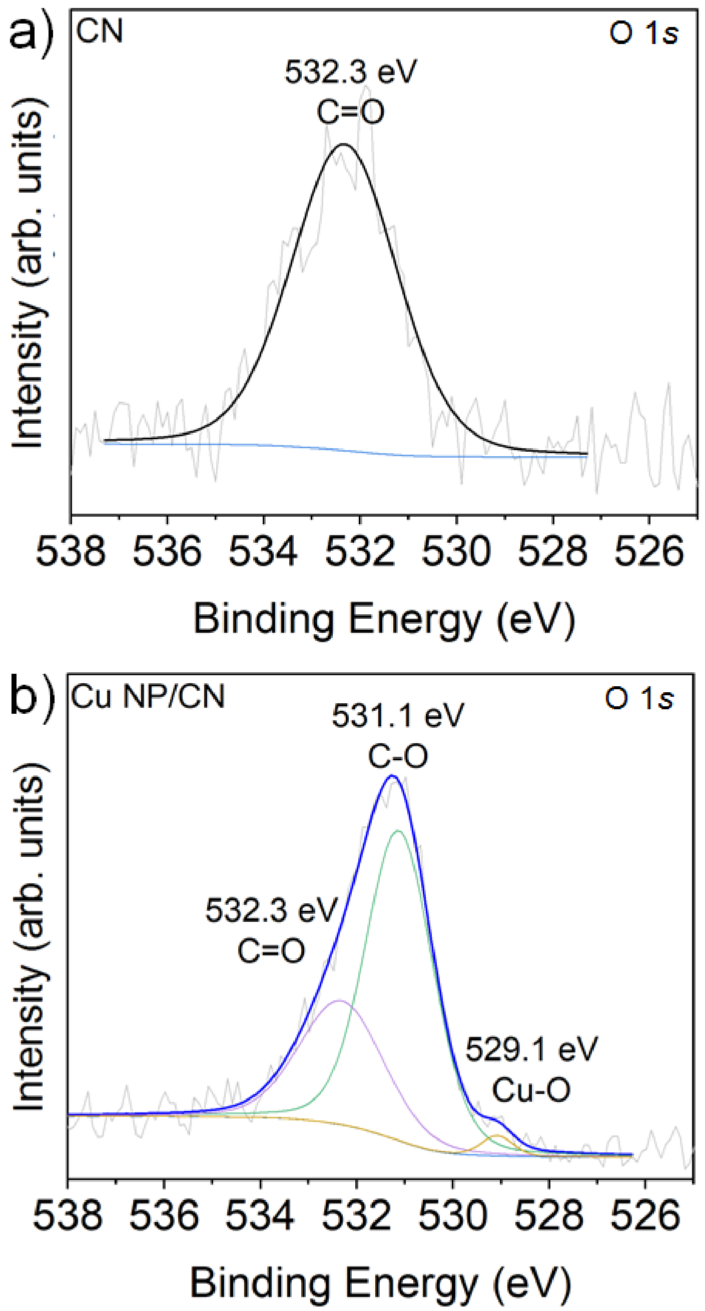


**Supplementary Fig. 9** The O 1s XPS spectra of a) CN and b) Cu NP/CN.

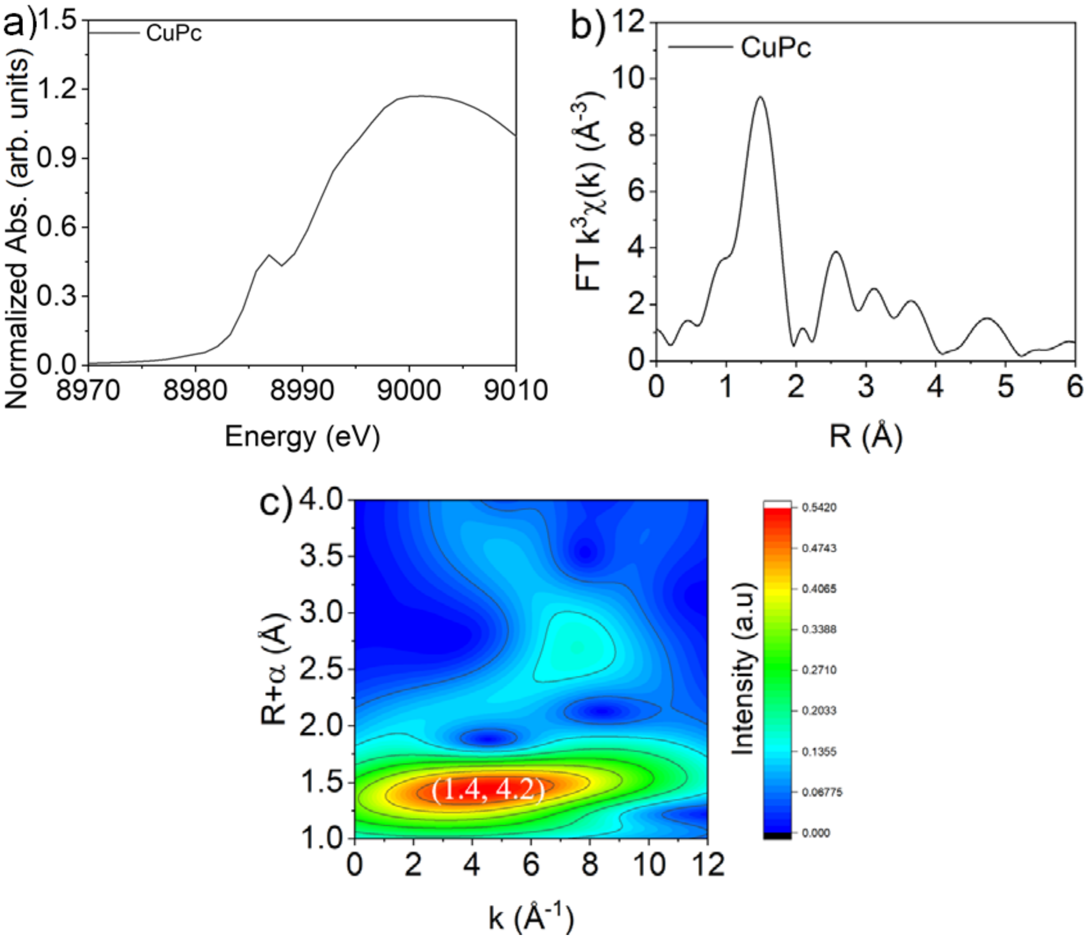


**Supplementary Fig. 10** a) Normalized Cu *K*-edge XANES spectrum, b) corresponding $k^3$-weighted Fourier Transform spectrum and c) Wavelet transform of CuPc.

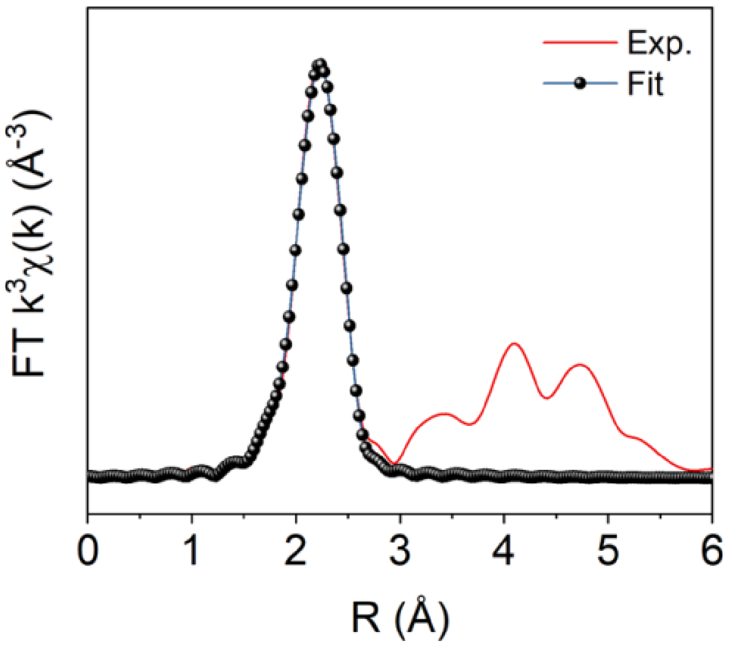


**Supplementary Fig. 11** EXAFS fitting curve in R space of Cu foil.

**Supplementary Table 4.** EXAFS fitting parameters at the Cu *K*-edge ($S_0^2$=0.86).

| Entry | Sample | Path | *C.N.* | *R* (Å) | $\sigma^2 \times 10^3$ (Å$^2$) | Δ*E* (eV) | *R* factor |
|---|---|---|---|---|---|---|---|
| 1 | Cu foil | Cu-Cu | 12* | 2.52±0.01 | 8.4±0.3 | 4.4±0.5 | 0.003 |
| 2 | Cu-$N_1O_2$ SA/CN | Cu-N(O) | 3.2±0.9 | 1.95±0.03 | 19.0±7.0 | 5.6±2.6 | 0.017 |

*C.N.*: coordination numbers; *R*: bond distance; $\sigma^2$: Debye-Waller factors; Δ*E*: the inner potential correction. *R* factor: goodness of fit. *fitting with fixed parameter.

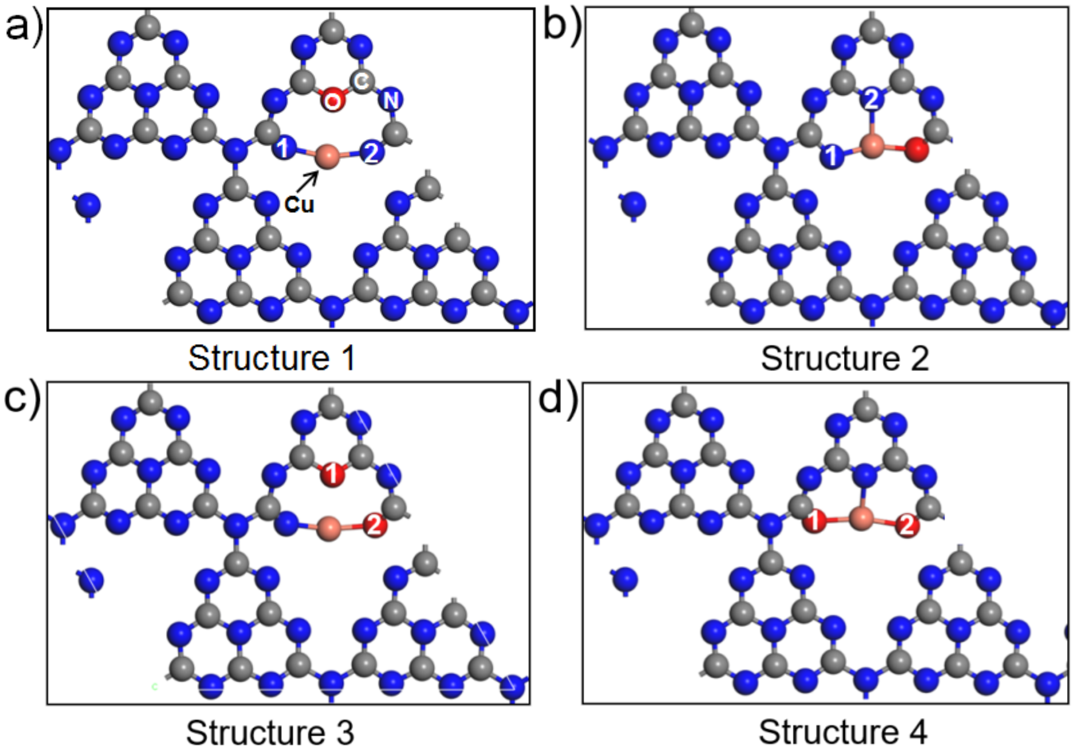


**Supplementary Fig. 12** The structure models of Cu-$N_xO_y$ SA/CN constructed by DFT calculation. a,b) $Cu_1$-$N_2O_1$ modle. c,d) $Cu_1$-$N_1O_2$ modle.

**Supplementary Table 5.** The parameters of proposed structures by DFT in Figure S12

| Structure | Bond distance (Å) | | Total energy (eV) |
|---|---|---|---|
| 1 | Cu-N1 | 1.76 | -464.8 |
| | Cu-N2 | 1.76 | |
| | Cu-O | 2.64 | |
| 2 | Cu-N1 | 1.75 | -466.8 |
| | Cu-N2 | 1.75 | |
| | Cu-O | 1.88 | |
| 3 | Cu-N | 1.76 | -464.7 |
| | Cu-O1 | 2.57 | |
| | Cu-O2 | 1.85 | |
| 4 | Cu-N | 1.91 | -466.4 |
| | Cu-O1 | 1.86 | |
| | Cu-O2 | 1.86 | |

**Supplementary Table 6.** The reported results of benzene selective oxidation to phenol.

| Entry | Catalyst | Reaction conditions | $H_2O_2$/benzene molar ratio | *Conv.* % | *Sel.* % | *TOF* $h^{-1}$ ($t^{[a]}$) | *Ref.* |
|---|---|---|---|---|---|---|---|
| 1 | $Cu_1$-$N_2$/HCNS | 20 mg of Cat., 6 mL MeCN, 0.5 mL benzene, 5.2 mL of $H_2O_2$(30%), 60 °C, 2.5 h | 10:1 | 70.9 | 91.1 | **2774 (2.5 h)** | 14 |
| 2 | Cu-SA/HCNS | 50 mg of Cat., 6 mL MeCN, 0.4 mL benzene, 6 mL of $H_2O_2$(30%), 60 °C, 12 h | 12:1 | 86 | 96.7 | 160 (1 h) | 15 |
| 3 | $FeN_4$/GN-2.7 | 50 mg of Cat., 3 mL MeCN, 0.4 mL benzene, 6 mL of $H_2O_2$(30%), 25 °C, 24 h | 12:1 | 23.4 | 81.9 | 24.3 (1 h) | 16 |
| 4 | SA-Fe/CN | 20 mg of Cat., 6 mL MeCN, 0.5 mL benzene, 5.2 mL of $H_2O_2$(30%), 60 °C, 24 h | 10:1 | 45 | 94 | 32.9 (24 h) | 17 |
| 5 | Co-ISA/CNS | 50 mg of Cat., 3 mL MeCN, 0.4 mL benzene, 6 mL of $H_2O_2$(30%), 25 °C, 96 h | 12:1 | 68 | 89.7 | 150 (1 h) | 18 |
| 6 | Cu SAC/S-N | 25 mg of Cat., 3 mL MeCN, 0.2 mL benzene, 3 mL of $H_2O_2$(30%), 25 °C, 24 h | 12:1 | 42.3 | 93.4 | 123 (1 h) | 19 |
| 7 | ISAS Fe/NPC | 50 mg of Cat., 3 mL MeCN, 0.1 mL benzene, 6 mL of $H_2O_2$(30%), 60 °C, 24 h | 48:1 | 42.6 | >99.9 | 1.3 (24 h) | 20 |
| 8 | $Cu_1$/NOC | 15 mg of Cat., 5 mL MeCN, 0.3 mL benzene, 5 mL of $H_2O_2$(30%), 60 °C, 24 h | 13:1 | 70.9 | 94.0 | 330 (1 h) | 21 |
| 9 | $Cu_1$/NC | 10 mg of Cat., 6 mL MeCN, 0.3 mL benzene, 10 mL of $H_2O_2$(30%), 60 °C, 48 h | 26:1 | 82 | 96 | 200 (1 h) | 22 |

[a] the time for *TOF* calculation.

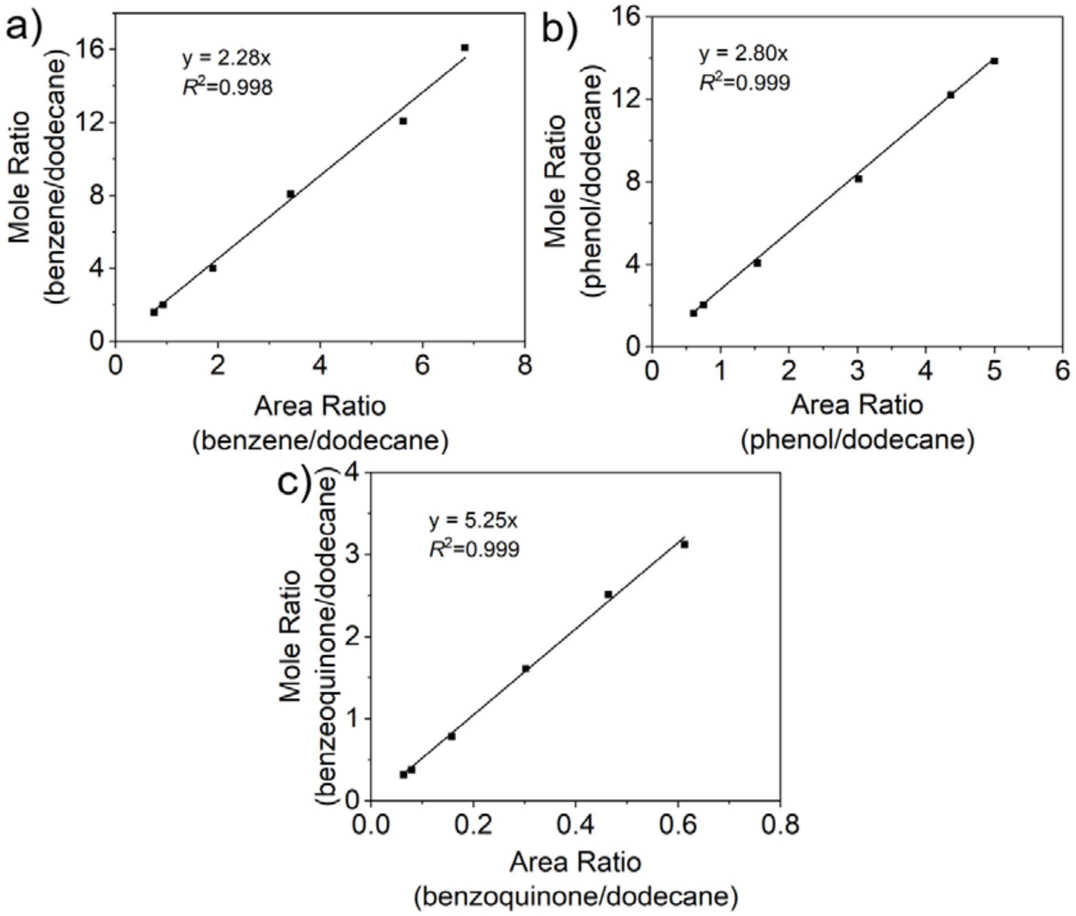


**Supplementary Fig. 13** The calibration curves of a) benzene, b) phenol and c) benzoquinone to dodecane for quantification.

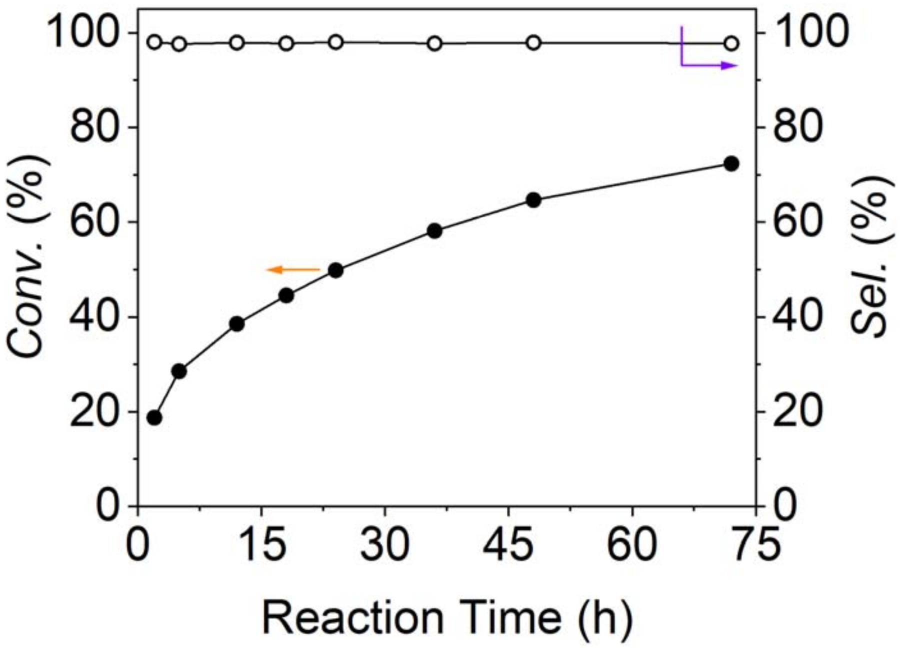


**Supplementary Fig. 14** The benzene conversion and phenol selectivity over Cu-$N_1O_2$ SA/CN under different reaction times. Reaction conditions: 30 mg catalyst, 0.4 mL benzene, 2:1 $H_2O_2$/benzene molar ratio, 2.0 mL $CH_3CN$ as solvent, 60 $^oC$.

**Supplementary Table 7.** Catalytic performance over various catalysts for SOBP.[a]

| Entry | Sample | Benzene conversion (%) | Selectivity (%) | | Phenol yield (%) | Carbon balance (%) |
|---|---|---|---|---|---|---|
| | | | Phenol | Benzoquinone | | |
| 1 | Blank | 0.2 | | | | |
| 2 | CN | 1.4 | 98.7 | 1.1 | 1.38 | 99.8 |
| 3 | Cu NP/CN | 13.5 | 74.6 | 10.8 | 10.1 | 85.4 |
| 4 | Cu-$N_1O_2$ SA/CN | 28.6 | 97.8 | 1.9 | 28.0 | 99.7 |
| 5 | Cu-$N_2$ SA/CN | 18.2 | 98.7 | 0.9 | 18.0 | 99.6 |
| 6 | Cu-$N_3$ SA/CN | 19.5 | 98.5 | 0.8 | 19.2 | 99.3 |
| 7[b] | Cu-$N_1O_2$ SA/CN | 83.7 | 98.1 | 0.9 | 82.1 | 99.0 |

[a] Reaction conditions: 30 mg, 0.4 mL benzene, 2:1$H_2O_2$/benzene molar ratio, 2.0 mL $CH_3CN$ as solvent, 60 $^oC$, 5 h.

[b] 50 mg catalyst, 72 h.

**Supplementary Table 8.** Reaction results of benzene selective oxidation to phenol over various samples.[a]

| Entry | Sample | Benzene conversion (%) | Phenol selectivity (%) | *TOF* ($h^{-1}$) | $H_2O_2$ conversion (%) | $H_2O_2$ utilization (%) |
|---|---|---|---|---|---|---|
| 1 | CN[b] | 1.4 | 99.7 | | | |
| 2 | CN[c] | 1.2 | 99.8 | | | |
| 3 | Cu NP/CN[b] | 13.5 | 74.6 | 58 | 74.3 | 9.1 |
| 4 | Cu NP/CN[c] | 10.9 | 79.3 | 65 | 58.6 | 9.3 |
| 5 | Cu-$N_2$ SA/CN[b] | 18.2 | 98.7 | 190 | 24.9 | 36.5 |
| 6 | Cu-$N_2$ SA/CN[c] | 23.6 | 96.8 | 215 | 31.0 | 38.0 |

[a] General reaction conditions: 30 mg of catalyst, 0.4 mL of benzene, $H_2O_2$/Benzene mole ratio is 2:1, 1 mL of $CH_3CN$ as solvent, 60 $^oC$, 5 h.

The reaction results show that Cu nanoparticles and Cu-$N_2$ moities supported on two kinds of CN matrixs display similar *TOF* value and phenol selectivity. Moreover, the two CN supports show a quite low benzene conversion. Therefore, the extrordinary performance of Cu-$N_1O_2$ SA/CN should be attributed to the unique $Cu_1$-$N_1O_2$ sites.

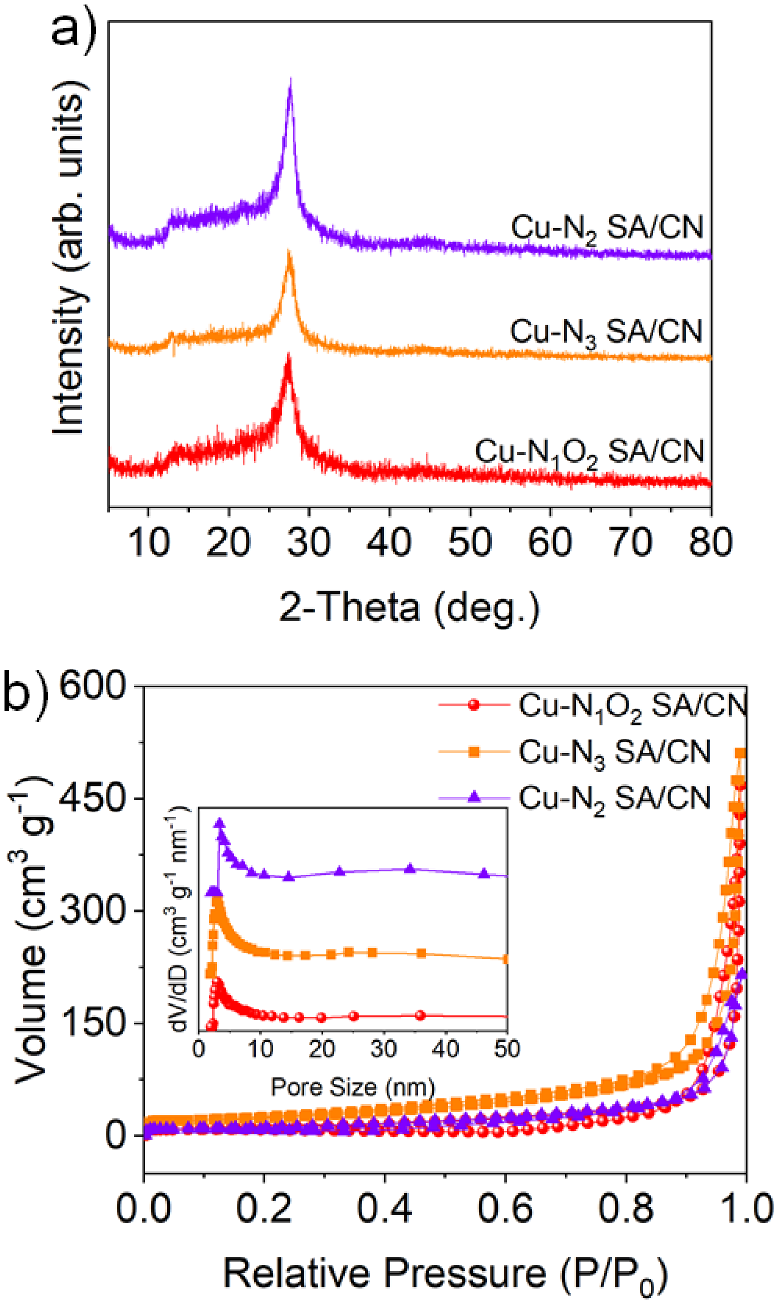


**Supplementary Fig. 15** Comparison of structure properties between single-atom Cu-$N_2$ SA/CN, Cu-$N_3$ SA/CN and Cu-$N_1O_2$ SA/CN. a) XRD patterns, b) $N_2$-phsysorption isothermal curve (insert: Pore size distributions).

**Supplementary Table 9.** Comparison of the textural properties of single-atom Cu-$N_2$ SA/CN, Cu-$N_3$ SA/CN and Cu-$N_1O_2$ SA/CN.

| Entry | Sample | $S_{BET}$ ($m^2$ $g^{-1}$) | $V_{BJH}$ ($cm^3$ $g^{-1}$) | Pore size (nm) |
|---|---|---|---|---|
| 1 | Cu-$N_2$ SA/CN | 42.2 | 0.37 | 3.2 |
| 2 | Cu-$N_3$ SA/CN | 84.0 | 0.82 | 3.0 |
| 3 | Cu-$N_1O_2$ SA/CN | 41.1 | 0.72 | 3.0 |

**Supplementary Table 10.** Comparison of the surface component of single-atom Cu-$N_2$ SA/CN, Cu-$N_3$ SA/CN and Cu-$N_1O_2$ SA/CN.

| Entry | Sample | C (at%) | N (at%) | O (at%) | C/N |
|---|---|---|---|---|---|
| 1 | Cu-$N_2$ SA/CN | 49.29 | 47.85 | 2.86 | 1.03 |
| 2 | Cu-$N_3$ SA/CN | 53.67 | 41.57 | 4.44 | 1.29 |
| 3 | Cu-$N_1O_2$ SA/CN | 49.14 | 49.07 | 1.79 | 1.00 |

**Supplementary Table 11.** The content of different N species of as-prepared samples.

| Entry | Sample | (-C=N-C-)+ (Cu-N) (%) | -N-(C)$_3$ (%) | -NH$_x$ (%) |
|---|---|---|---|---|
| 1 | CN | 76 | 18 | 6 |
| 2 | Cu-$N_1O_2$ SA/CN | 80 | 13 | 7 |
| 3 | Cu-$N_2$ SA/CN | 80 | 9 | 11 |
| 4 | Cu-$N_3$ SA/CN | 73 | 22 | 5 |

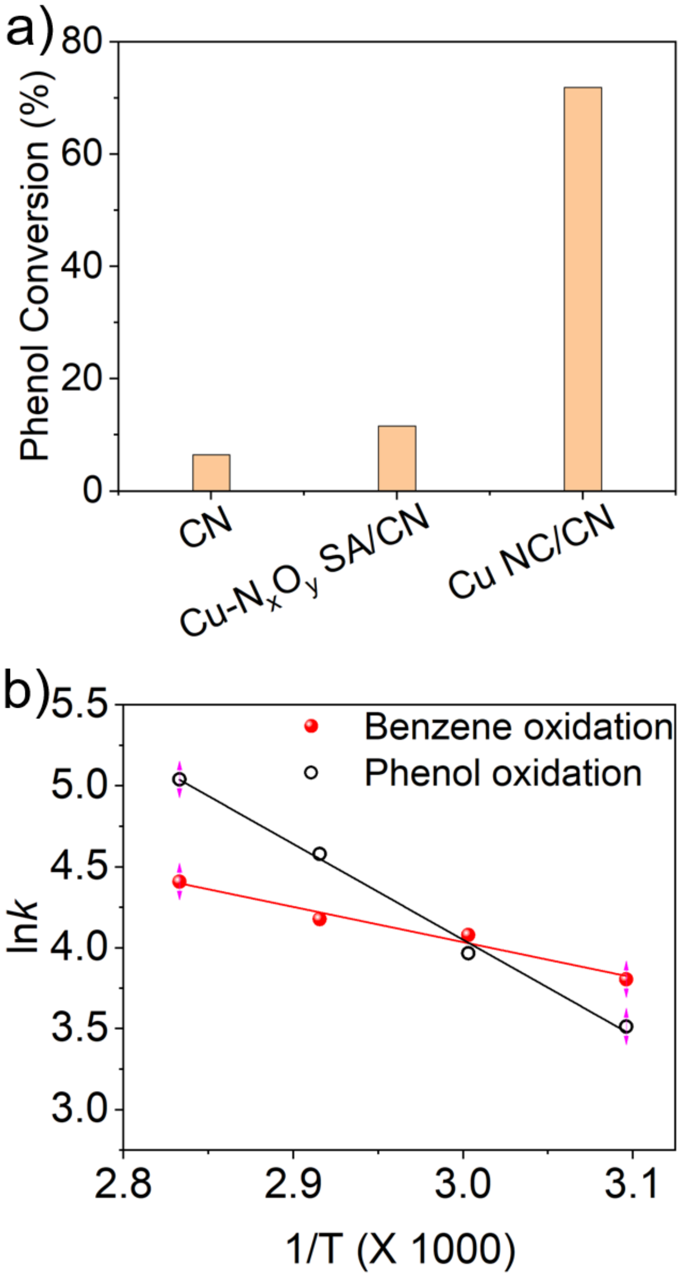


**Supplementary Fig. 16** a) Phenol oxidation with $H_2O_2$ catalysed with CN, Cu-$N_1O_2$ SA/CN and Cu NP/CN, respectively. b) Arrhenius plots for benzene oxidation and phenol oxidation over Cu-$N_1O_2$ SA/CN.

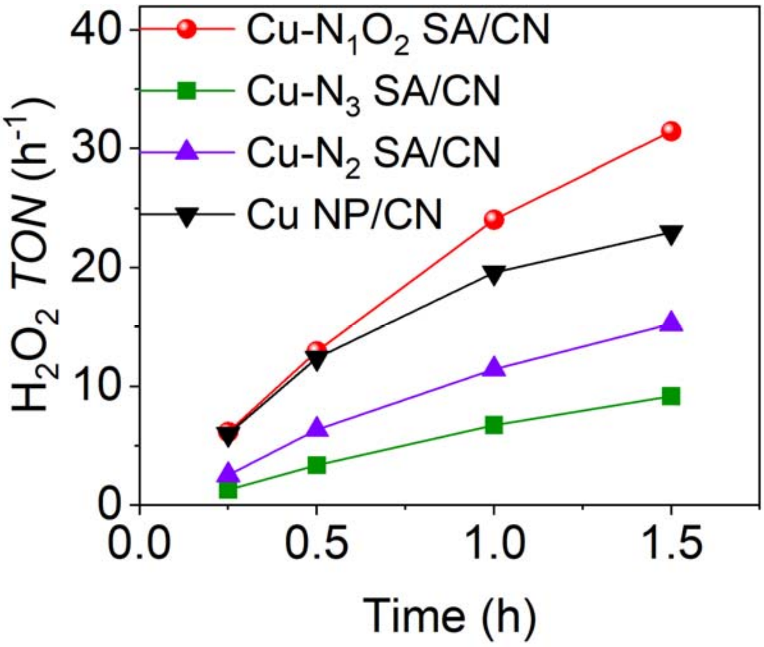


**Supplementary Fig. 17** The comparison of $H_2O_2$ activation over various catalysts. Reaction conditions: 10 mg of catalyst, 2.0 mL of benzene, 1:10 $H_2O_2$/benzene molar ratio, 2.0 mL of $CH_3CN$ as solvent, 60 $^oC$.

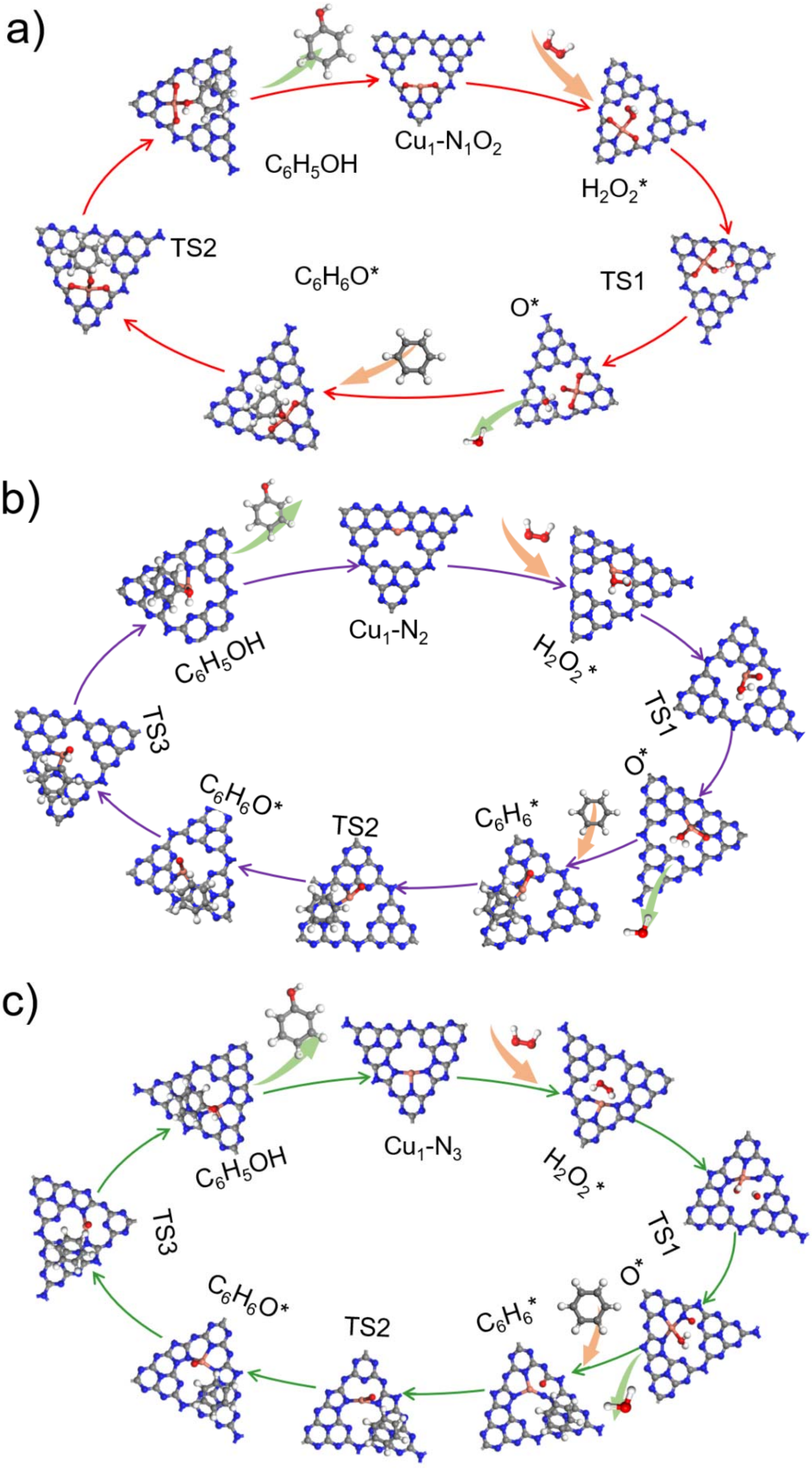


**Supplementary Fig. 18** Reaction pathway of $H_2O_2$ oxidize benzene to phenol over single-atom a) $Cu_1$-$N_1O_2$ site, b) $Cu_1$-$N_2$ site and c) $Cu_1$-$N_3$ site. TS: transient state.

**Supplementary Table 12.** The adsorption energy ($E_{ads}$) of benzene and desorption energy ($E_{des}$) of phenol over $Cu_1$-$N_2$, $Cu_1$-$N_3$ and $Cu_1$-$N_1O_2$ sites in the presence of $CH_3CN$ solvent.

| Catalysts | $E_{ads}$ (benzene) | $E_{des}$ (phenol) |
|---|---|---|
| Cu-$N_1O_2$ SA/CN | -1.17 | 1.65 |
| Cu-$N_2$ SA/CN | -1.49 | 1.53 |
| Cu-$N_3$ SA/CN | -1.17 | 1.31 |

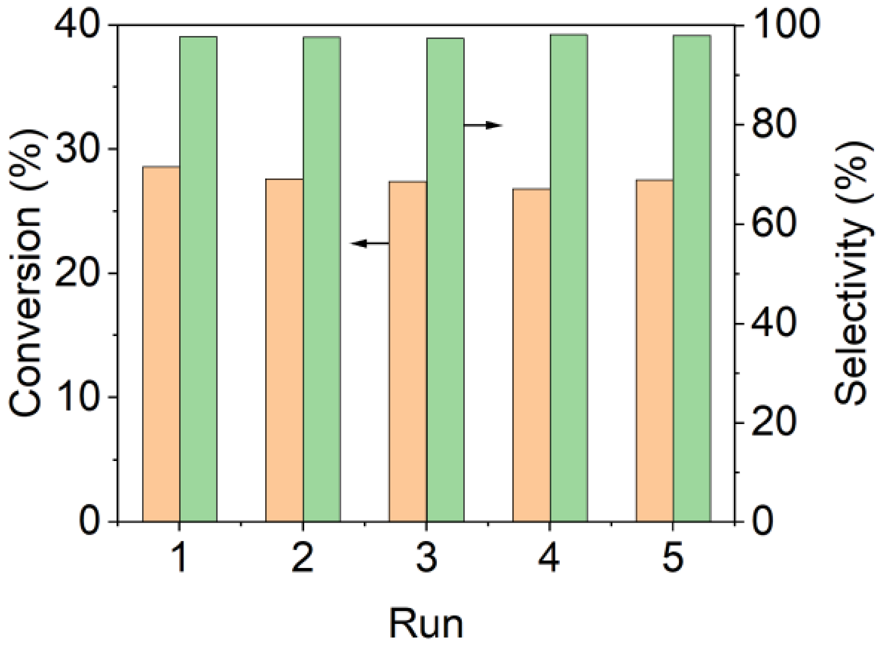


**Supplementary Fig. 19** Recycalbility of single-atom Cu-$N_1O_2$ SA/CN catalyst for benzene oxidation to phenol.

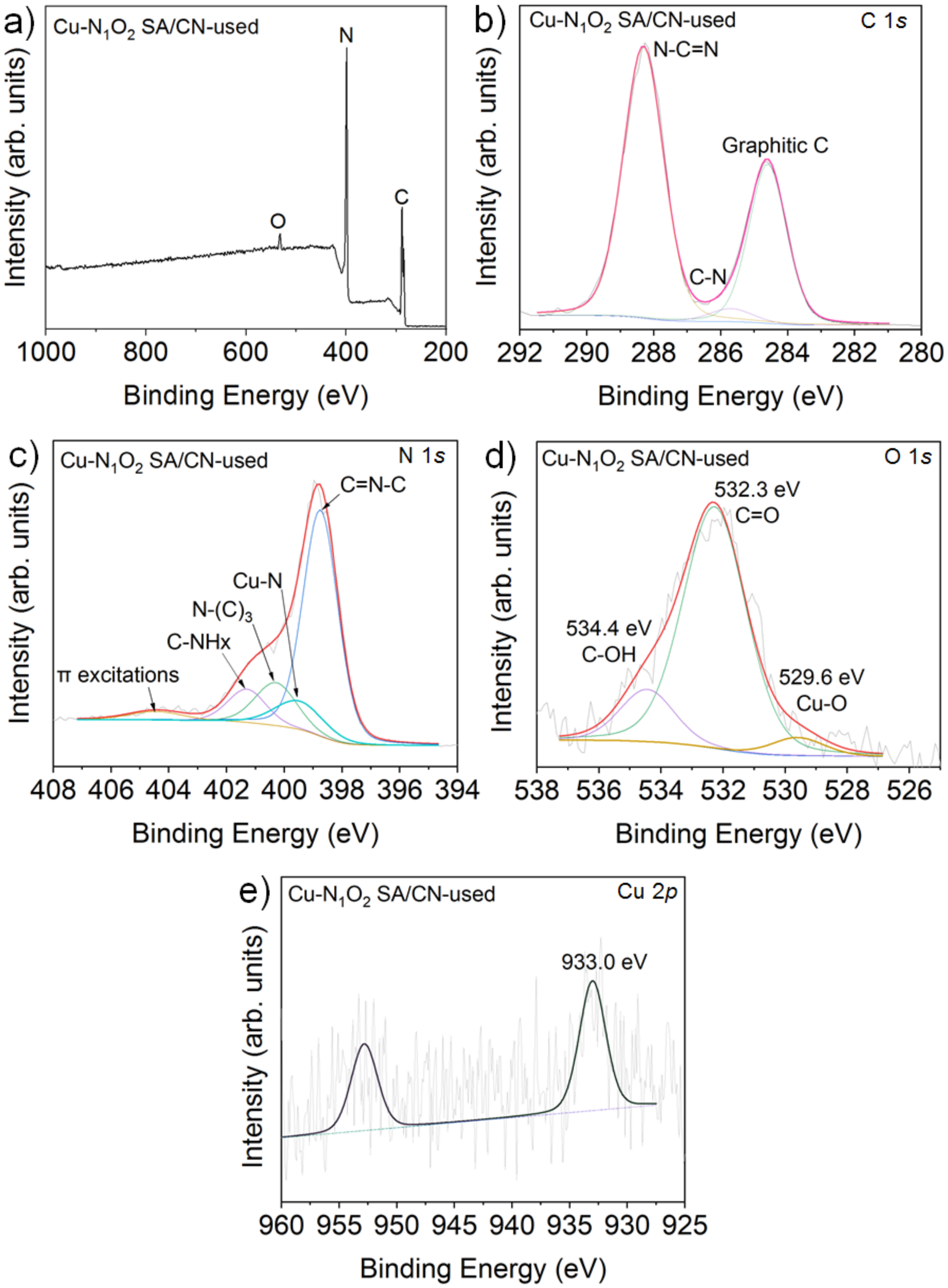


**Supplementary Fig. 20** XPS characterization of the used single-atom catalyst $Cu-N_1O_2$ SA/CN-used. a) The survey XPS spectrum. b) The C 1*s* spectrum. c) The N 1*s* spectrum. d) The O 1*s* spectrum. e) The Cu 2*p* spectrum.

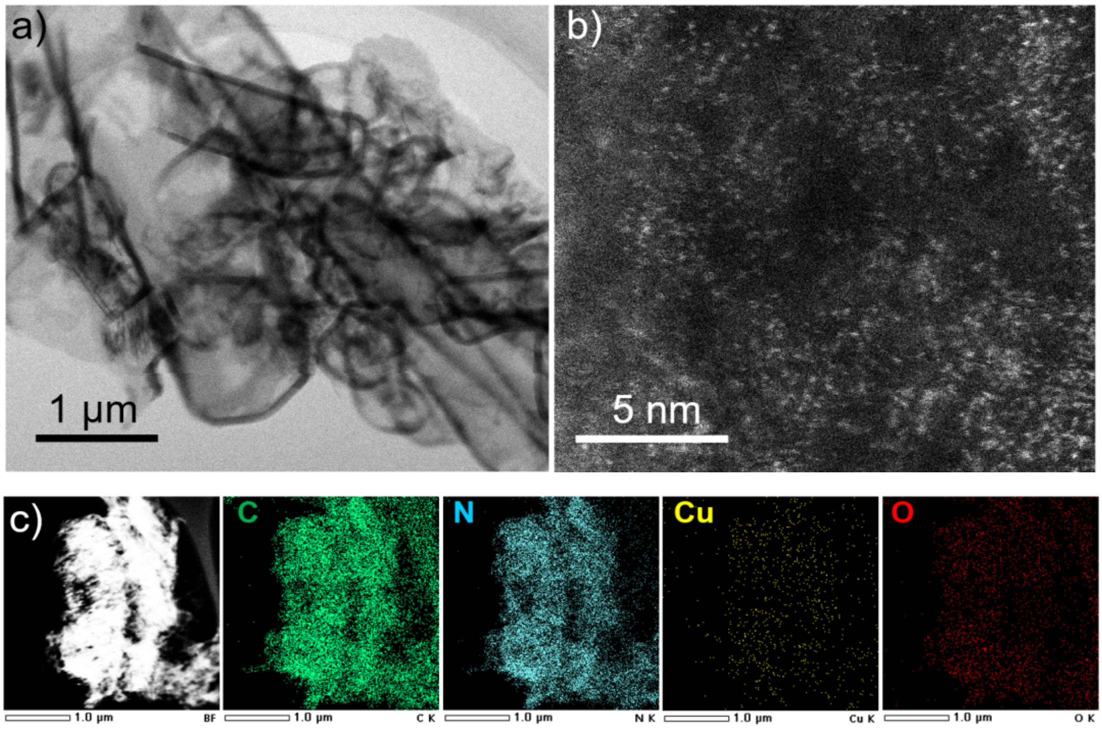


**Supplementary Fig. 21** (a) TEM image, (b) HAADF-STEM images and (c) elemental EDS mapping of the used single-atom catalyst Cu-$N_1O_2$ SA/CN-used.

**Supplementary references**


(1) Delley, B. From molecules to solids with the DMol3 approach. *J. Chem. Phys.* **113**, 7756−7764 (2000).

(2) Delley, B. An all-electron numerical method for solving the local density functional for polyatomic molecules. *J. Chem. Phys.* **92**, 508−517 (1990).

(3) Perdew, J. P.; Chevary, J. A.; Vosko, S. H.; Jackson, Koblar, A.; Pederson, M. R.; Singh, D. J.; Fiolhais, C. Atoms, molecules, solids, and surfaces: applications of the generalized gradient approximation for exchange and correlation. *Phys. Rev. B* **46**, 6671−6687 (1992).

(4) Perdew, J. P.; Burke, K.; Ernzerhof, M. Generalized gradient approximation made simple. *Phys. Rev. Lett.* **77**, 3865−3868 (1996).

(5) Hammer, B.; Hansen, L. B.; Nørskov, J. K. Improved adsorption energetics within density-functional theory using revised perdew-burke-ernzerhof functionals. *Phys. Rev. B* **59**, 7413−7421 (1999).

(6) Dolg, M.; Wedig, U.; Stoll, H.; Preuss, H. Energy-adjusted ab initio pseudopotentials for the first row transition elements. *J. Chem. Phys.* **86**, 866−872 (1987).

(7) Tian, D. X.; Zhang, H. L.; Zhao, J. J. Structure and structural evolution of $Ag_n$ (n=3-22) clusters using a genetic algorithm and density functional theory method. *Solid State Commun.* **144**, 174−179 (2007).

(8) Wang, L.; Lu, Q. Q.; Zhang, W. H.; Yang, J. L. Transition metal atom embedded graphene for capturing CO: a first-principles study. *Int. J. Hydrogen Energy* **39**, 20190−20196 (2014).

(9) Esrafili, M. D.; Nejadebrahimi, B. Theoretical insights into hydrogenation of $CO_2$ to formic acid over a single Co atom incorporated nitrogen-doped graphene: a DFT study. *Appl. Surf. Sci.* **475**, 363−371 (2019).

(10) Halgren, T. A.; Lipscomb, W. N. The synchronous-transit method for determining reaction pathways and locating molecular transition states. *Chem. Phys. Lett.* **49**, 225−232 (1977).

(11) Govind, N.; Petersen, M.; Fitzgerald, G.; King-Smith, D.; Andzelm, J. A generalized synchronous transit method for transition state location. *Comput. Mater. Sci.* **28**, 250−258 (2003).

(12) Matthew, S. T.; Jeanne, M. H.; Andrew, A. G. Poisoning the oxygen reduction reaction on carbon-supported Fe and Cu electrocatalysts: evidence for metal-centered activity. *J. Phys. Chem. Lett.* **2**, 295–298 (2011).

(13) Matthew, S. T.; Jeanne, M. H.; Andrew, A. G. Discriminating catalytically active $FeN_x$ species of atomically dispersed Fe–N–C catalyst for selective oxidation of the C–H bond. *J. Am. Chem. Soc.* **139,** 10790–10798 (2017).

(14) Zhang, T.; Zhang, Di; Han, X.; Dong, T.; Guo, X.; Song, C.; Si, Rui; L., Wei; Liu, Y.; Zhao, Z. Preassembly strategy to fabricate porous hollow carbonitride spheres inlaid with single $Cu–N_3$ sites for selective oxidation of benzene to phenol. *J. Am. Chem. Soc.***140**, 16936–16940 (2018).

(15) Zhang, T.; Nie, X.; Yu, W.; Guo, X.; Song, C.; Si, R.; Liu, Y.; Zhao, Z. Single atomic $Cu-N_2$ catalytic sites for highly active and selective hydroxylation of benzene to phenol. *iScience* **22**, 97-108 (2019).

(16) Deng, D.; Chen, X.; Yu, L.; Wu, X.; Liu, Q.; Liu, Y.; Yang, H.; Tian, H.; Hu, Y.; Du, P.; Si, R.; Wang, J.; Cui, X.; Li, H.; Xiao, J.; Xu, T.; Deng, J.; Yang, F.; Duchesne, P. N.; Zhang, P.; Zhou, J.; Sun, L.; Li, J.; Pan, X.; Bao, X. A single iron site confined in a graphene matrix for the catalytic oxidation of benzene at room temperature. *Sci. Adv.* **1**, e150046 (2015).

(17) Zhang, M.; Wang, Y.-G.; Chen, W.; Dong, J.; Zheng, L.; Luo, J.; Wan, J.; Tian, S.; Cheong, W.-C.; Wang, D.; Li, Y. Metal (hydr)oxides@polymer core–shell strategy to metal single-atom materials. *J. Am. Chem. Soc.* **139**, 10976–10979 (2017).

(18) Zhu, Y.; Sun, W.; Luo, J.; Chen, W.; Cao, T.; Zheng, L.; Dong, J.; Zhang, J.; Zhang, M.; Han, Y.; Chen, C.; Peng, Q.; Wang, D.; Li, Y. A cocoon silk chemistry strategy to ultrathin N-doped carbon nanosheet with metal single-site catalysts, *Nat. Commun.* **9**, 3861 (2018).

(19) Zhou, H.; Zhao, Y.; Gan, J.; Xu, J.; Wang, Y.; Lv, H.; Fang, S.; Wang, Z.; Deng, Z.; Wang, X.; Liu, P.; Guo, W.; Mao, B.; Wang, H.; Yao, T.; Hong, X.; Wei, S.; Duan, X.; Luo, J.; Wu, Y. Cation-exchange induced precise regulation of single copper site triggers room-temperature oxidation of benzene. *J. Am. Chem. Soc.* **142**, 12643–12650 (2020).

(20) Wu, K.; Zhan, F.; Tu, R.; Cheong, W.-C.; Cheng, Y.; Zheng, L.; Yan, W.; Zhang, Q.; Chen, Z.; Chen, C. Dopamine polymer derived isolated single-atom site metals/N-doped porous carbon for benzene oxidation. *Chem. Commun.* **56**, 8916-8919 (2020).

(21) Chen, W.; Jin, H.; He, F.; Cui, P.; Cao, C.; Song, W. Dynamic evolution of nitrogen and oxygen dual-coordinated single atomic copper catalyst during partial oxidation of benzene to phenol. *Nano Res.* **15**, 3017–3025 (2021).

(22) Shen, Q.; Li, P.; Chen, W.; Jin, H.; Yu, J.; Zhu, L.; Yang, Z.; Zhao, R.; Zheng, L.; Song, W.; Cao, C. Ionic-liquid-assisted synthesis of metal single-atom catalysts for benzene oxidation to phenol. *Sci. China Mater.* **65**, 163–169 (2022).
